# Supplementary material for: HIVprotI: an integrated web based platform for prediction and design of HIV proteins inhibitors
Source: J Cheminform. 2018 Mar 9;10:12. doi: 10.1186/s13321-018-0266-y (PMC5845081; doi:10.1186/s13321-018-0266-y)
Supplement: Supplementary file 2 — Additional file 2. Source code of HIVProtI web server. [file 13321_2018_266_MOESM2_ESM.zip › HIVprotI_Source-code/hivprotI/weka-3-6-6/doc/weka/filters/unsupervised/instance/RemoveFrequentValues.html]

RemoveFrequentValues


---


|  |  |  |
| --- | --- | --- |
| **Overview** \| **Package** \| **Class** \| **Tree** \| **Deprecated** \| **Index** \| **Help** \| **Weka's home** | |  |
| **PREV CLASS**   **NEXT CLASS** | **FRAMES**    **NO FRAMES**     **All Classes** |  |
| SUMMARY: NESTED \| FIELD \| CONSTR \| METHOD | DETAIL: FIELD \| CONSTR \| METHOD |  |


---


## weka.filters.unsupervised.instance Class RemoveFrequentValues

```
java.lang.Object
  weka.filters.Filter
      weka.filters.unsupervised.instance.RemoveFrequentValues
```

**All Implemented Interfaces:**: java.io.Serializable, CapabilitiesHandler, OptionHandler, RevisionHandler, UnsupervisedFilter

---

``` public class RemoveFrequentValues extends Filter implements OptionHandler, UnsupervisedFilter ```

Determines which values (frequent or infrequent ones) of an (nominal) attribute are retained and filters the instances accordingly. In case of values with the same frequency, they are kept in the way they appear in the original instances object. E.g. if you have the values "1,2,3,4" with the frequencies "10,5,5,3" and you chose to keep the 2 most common values, the values "1,2" would be returned, since the value "2" comes before "3", even though they have the same frequency.


Valid options are:

```
 -C <num>
  Choose attribute to be used for selection.
```

```
 -N <num>
  Number of values to retain for the sepcified attribute, 
  i.e. the ones with the most instances (default 2).
```

```
 -L
  Instead of values with the most instances the ones with the 
  least are retained.
```

```
 -H
  When selecting on nominal attributes, removes header
  references to excluded values.
```

```
 -V
  Invert matching sense.
```

**Version:**
:   $Revision: 5548 $

**Author:**
:   FracPete (fracpete at waikato dot ac dot nz)

**See Also:**: Serialized Form

---

**Constructor Summary**

`RemoveFrequentValues()`


| **Method Summary** | |
| --- | --- |
| `java.lang.String` | `attributeIndexTipText()`             Returns the tip text for this property |
| `boolean` | `batchFinished()`             Signifies that this batch of input to the filter is finished. |
| `void` | `determineValues(Instances inst)`             determines the values to retain, it is always at least 1 and up to the maximum number of distinct values |
| `java.lang.String` | `getAttributeIndex()`             Get the index of the attribute used. |
| `Capabilities` | `getCapabilities()`             Returns the Capabilities of this filter. |
| `boolean` | `getInvertSelection()`             Get whether the supplied columns are to be removed or kept |
| `boolean` | `getModifyHeader()`             Gets whether the header will be modified when selecting on nominal attributes. |
| `int` | `getNumValues()`             Gets how many values are retained |
| `java.lang.String[]` | `getOptions()`             Gets the current settings of the filter. |
| `java.lang.String` | `getRevision()`             Returns the revision string. |
| `boolean` | `getUseLeastValues()`             Gets whether to use values with least or most instances |
| `java.lang.String` | `globalInfo()`             Returns a string describing this filter |
| `boolean` | `input(Instance instance)`             Input an instance for filtering. |
| `java.lang.String` | `invertSelectionTipText()`             Returns the tip text for this property |
| `boolean` | `isNominal()`             Returns true if selection attribute is nominal. |
| `java.util.Enumeration` | `listOptions()`             Returns an enumeration describing the available options. |
| `static void` | `main(java.lang.String[] argv)`             Main method for testing this class. |
| `java.lang.String` | `modifyHeaderTipText()`             Returns the tip text for this property |
| `java.lang.String` | `numValuesTipText()`             Returns the tip text for this property |
| `void` | `setAttributeIndex(java.lang.String attIndex)`             Sets index of the attribute used. |
| `boolean` | `setInputFormat(Instances instanceInfo)`             Sets the format of the input instances. |
| `void` | `setInvertSelection(boolean invert)`             Set whether selected values should be removed or kept. |
| `void` | `setModifyHeader(boolean newModifyHeader)`             Sets whether the header will be modified when selecting on nominal attributes. |
| `void` | `setNumValues(int numValues)`             Sets how many values are retained |
| `void` | `setOptions(java.lang.String[] options)`             Parses a given list of options. |
| `void` | `setUseLeastValues(boolean leastValues)`             Sets whether to use values with least or most instances |
| `java.lang.String` | `useLeastValuesTipText()`             Returns the tip text for this property |

**Methods inherited from class weka.filters.Filter**

`batchFilterFile, filterFile, getCapabilities, getOutputFormat, isFirstBatchDone, isNewBatch, isOutputFormatDefined, makeCopies, makeCopy, numPendingOutput, output, outputPeek, toString, useFilter, wekaStaticWrapper`

**Methods inherited from class java.lang.Object**

`equals, getClass, hashCode, notify, notifyAll, wait, wait, wait`

**Constructor Detail**

### RemoveFrequentValues

```
public RemoveFrequentValues()
```


**Method Detail**

### globalInfo

```
public java.lang.String globalInfo()
```

:   Returns a string describing this filter

    :   **Returns:**: a description of the classifier suitable for displaying in the explorer/experimenter gui

---


### listOptions

```
public java.util.Enumeration listOptions()
```

:   Returns an enumeration describing the available options.

    :   **Specified by:**: `listOptions` in interface `OptionHandler`
    :   **Returns:**: an enumeration of all the available options.

---


### setOptions

```
public void setOptions(java.lang.String[] options)
                throws java.lang.Exception
```

:   Parses a given list of options.


    Valid options are:

    ```
     -C <num>
      Choose attribute to be used for selection.
    ```

    ```
     -N <num>
      Number of values to retain for the sepcified attribute, 
      i.e. the ones with the most instances (default 2).
    ```

    ```
     -L
      Instead of values with the most instances the ones with the 
      least are retained.
    ```

    ```
     -H
      When selecting on nominal attributes, removes header
      references to excluded values.
    ```

    ```
     -V
      Invert matching sense.
    ```

    :   **Specified by:**: `setOptions` in interface `OptionHandler`
    :   **Parameters:**: `options` - the list of options as an array of strings **Throws:**: `java.lang.Exception` - if an option is not supported

---


### getOptions

```
public java.lang.String[] getOptions()
```

:   Gets the current settings of the filter.

    :   **Specified by:**: `getOptions` in interface `OptionHandler`
    :   **Returns:**: an array of strings suitable for passing to setOptions

---


### attributeIndexTipText

```
public java.lang.String attributeIndexTipText()
```

:   Returns the tip text for this property

    :   **Returns:**: tip text for this property suitable for displaying in the explorer/experimenter gui

---


### getAttributeIndex

```
public java.lang.String getAttributeIndex()
```

:   Get the index of the attribute used.

    :   **Returns:**: the index of the attribute

---


### setAttributeIndex

```
public void setAttributeIndex(java.lang.String attIndex)
```

:   Sets index of the attribute used.

    :   **Parameters:**: `attIndex` - the index of the attribute

---


### numValuesTipText

```
public java.lang.String numValuesTipText()
```

:   Returns the tip text for this property

    :   **Returns:**: tip text for this property suitable for displaying in the explorer/experimenter gui

---


### getNumValues

```
public int getNumValues()
```

:   Gets how many values are retained

    :   **Returns:**: how many values are retained

---


### setNumValues

```
public void setNumValues(int numValues)
```

:   Sets how many values are retained

    :   **Parameters:**: `numValues` - the number of values to retain

---


### useLeastValuesTipText

```
public java.lang.String useLeastValuesTipText()
```

:   Returns the tip text for this property

    :   **Returns:**: tip text for this property suitable for displaying in the explorer/experimenter gui

---


### getUseLeastValues

```
public boolean getUseLeastValues()
```

:   Gets whether to use values with least or most instances

    :   **Returns:**: true if values with least instances are retained

---


### setUseLeastValues

```
public void setUseLeastValues(boolean leastValues)
```

:   Sets whether to use values with least or most instances

    :   **Parameters:**: `leastValues` - whether values with least or most instances are retained

---


### modifyHeaderTipText

```
public java.lang.String modifyHeaderTipText()
```

:   Returns the tip text for this property

    :   **Returns:**: tip text for this property suitable for displaying in the explorer/experimenter gui

---


### getModifyHeader

```
public boolean getModifyHeader()
```

:   Gets whether the header will be modified when selecting on nominal
    attributes.

    :   **Returns:**: true if so.

---


### setModifyHeader

```
public void setModifyHeader(boolean newModifyHeader)
```

:   Sets whether the header will be modified when selecting on nominal
    attributes.

    :   **Parameters:**: `newModifyHeader` - true if so.

---


### invertSelectionTipText

```
public java.lang.String invertSelectionTipText()
```

:   Returns the tip text for this property

    :   **Returns:**: tip text for this property suitable for displaying in the explorer/experimenter gui

---


### getInvertSelection

```
public boolean getInvertSelection()
```

:   Get whether the supplied columns are to be removed or kept

    :   **Returns:**: true if the supplied columns will be kept

---


### setInvertSelection

```
public void setInvertSelection(boolean invert)
```

:   Set whether selected values should be removed or kept. If true the
    selected values are kept and unselected values are deleted.

    :   **Parameters:**: `invert` - the new invert setting

---


### isNominal

```
public boolean isNominal()
```

:   Returns true if selection attribute is nominal.

    :   **Returns:**: true if selection attribute is nominal

---


### determineValues

```
public void determineValues(Instances inst)
```

:   determines the values to retain, it is always at least 1
    and up to the maximum number of distinct values

    :   **Parameters:**: `inst` - the Instances to determine the values from which are kept

---


### getCapabilities

```
public Capabilities getCapabilities()
```

:   Returns the Capabilities of this filter.

    :   **Specified by:**: `getCapabilities` in interface `CapabilitiesHandler` **Overrides:**: `getCapabilities` in class `Filter`
    :   **Returns:**: the capabilities of this object **See Also:**: `Capabilities`

---


### setInputFormat

```
public boolean setInputFormat(Instances instanceInfo)
                       throws java.lang.Exception
```

:   Sets the format of the input instances.

    :   **Overrides:**: `setInputFormat` in class `Filter`
    :   **Parameters:**: `instanceInfo` - an Instances object containing the input instance structure (any instances contained in the object are ignored - only the structure is required). **Returns:**: true if the outputFormat can be collected immediately **Throws:**: `UnsupportedAttributeTypeException` - if the specified attribute is not nominal.: `java.lang.Exception` - if the inputFormat can't be set successfully

---


### input

```
public boolean input(Instance instance)
```

:   Input an instance for filtering. Ordinarily the instance is processed
    and made available for output immediately. Some filters require all
    instances be read before producing output.

    :   **Overrides:**: `input` in class `Filter`
    :   **Parameters:**: `instance` - the input instance **Returns:**: true if the filtered instance may now be collected with output(). **Throws:**: `java.lang.IllegalStateException` - if no input format has been set.

---


### batchFinished

```
public boolean batchFinished()
```

:   Signifies that this batch of input to the filter is finished. If the
    filter requires all instances prior to filtering, output() may now
    be called to retrieve the filtered instances.

    :   **Overrides:**: `batchFinished` in class `Filter`
    :   **Returns:**: true if there are instances pending output **Throws:**: `java.lang.IllegalStateException` - if no input structure has been defined

---


### getRevision

```
public java.lang.String getRevision()
```

:   Returns the revision string.

    :   **Specified by:**: `getRevision` in interface `RevisionHandler` **Overrides:**: `getRevision` in class `Filter`
    :   **Returns:**: the revision

---


### main

```
public static void main(java.lang.String[] argv)
```

:   Main method for testing this class.

    :   **Parameters:**: `argv` - should contain arguments to the filter: use -h for help


---


|  |  |  |
| --- | --- | --- |
| **Overview** \| **Package** \| **Class** \| **Tree** \| **Deprecated** \| **Index** \| **Help** \| **Weka's home** | |  |
| **PREV CLASS**   **NEXT CLASS** | **FRAMES**    **NO FRAMES**     **All Classes** |  |
| SUMMARY: NESTED \| FIELD \| CONSTR \| METHOD | DETAIL: FIELD \| CONSTR \| METHOD |  |


---
